# Brain oscillations in reflecting motor status and recovery induced by action observation-driven robotic hand intervention in chronic stroke

**DOI:** 10.3389/fnins.2023.1241772

**Published:** 2023-12-11

**Authors:** Zan Yue, Peng Xiao, Jing Wang, Raymond Kai-yu Tong

**Affiliations:** ^1^Institute of Robotics and Intelligent Systems, Xi’an Jiaotong University, Xi’an, China; ^2^Neurorehabilitation Robotics Research Institute, Xi’an Jiaotong University, Xi’an, China; ^3^Department of Biomedical Engineering, The Chinese University of Hong Kong, Hong Kong, Hong Kong SAR, China

**Keywords:** chronic stroke, motor rehabilitation, biomarkers, brain-computer interface, electroencephalography, action observation

## Abstract

Hand rehabilitation in chronic stroke remains challenging, and finding markers that could reflect motor function would help to understand and evaluate the therapy and recovery. The present study explored whether brain oscillations in different electroencephalogram (EEG) bands could indicate the motor status and recovery induced by action observation-driven brain–computer interface (AO-BCI) robotic therapy in chronic stroke. The neurophysiological data of 16 chronic stroke patients who received 20-session BCI hand training is the basis of the study presented here. Resting-state EEG was recorded during the observation of non-biological movements, while task-stage EEG was recorded during the observation of biological movements in training. The motor performance was evaluated using the Action Research Arm Test (ARAT) and upper extremity Fugl–Meyer Assessment (FMA), and significant improvements (*p* < 0.05) on both scales were found in patients after the intervention. Averaged EEG band power in the affected hemisphere presented negative correlations with scales pre-training; however, no significant correlations (*p* > 0.01) were found both in the pre-training and post-training stages. After comparing the variation of oscillations over training, we found patients with good and poor recovery presented different trends in delta, low-beta, and high-beta variations, and only patients with good recovery presented significant changes in EEG band power after training (delta band, *p* < 0.01). Importantly, motor improvements in ARAT correlate significantly with task EEG power changes (low-beta, c.c = 0.71, *p* = 0.005; high-beta, c.c = 0.71, *p* = 0.004) and task/rest EEG power ratio changes (delta, c.c = −0.738, *p* = 0.003; low-beta, c.c = 0.67, *p* = 0.009; high-beta, c.c = 0.839, *p* = 0.000). These results suggest that, in chronic stroke, EEG band power may not be a good indicator of motor status. However, ipsilesional oscillation changes in the delta and beta bands provide potential biomarkers related to the therapeutic-induced improvement of motor function in effective BCI intervention, which may be useful in understanding the brain plasticity changes and contribute to evaluating therapy and recovery in chronic-stage motor rehabilitation.

## 1 Introduction

Stroke has been the leading cause of acquired disability in adults globally for decades (Mendis, 2013). Although the mortality rate declined with improved healthcare, approximately 80% of stroke victims still experience motor impairment, and more than 30% of patients suffer despite intensive rehabilitation (Lai et al., 2002; Young and Forster, 2007). It is worse for the chronic group with severe motor impairments in the upper limbs. On the one hand, effective interventions like constraint-induced movement therapy (CIMT) may not be applicable to those patients without enough residual active movement (Thrasher et al., 2008). On the other hand, motor recovery in chronic stroke is more challenging due to the decreasing plasticity of spontaneous recovery (Cassidy and Cramer, 2017). Since the upper limbs, especially the hands, play a significant role in daily activity, exploring novel rehabilitation therapies for hand motor recovery in this group is essential (Neumann, 2016). Robot-assisted therapy (RAT) and motor imagery (MI) have been introduced to enhance motor recovery for stroke patients through passive motion or mental practice. However, although these interventions benefit training without requiring patients’ residual ability, rehabilitation effectiveness is still limited by a lack of active engagement (Kwakkel et al., 2008; Ietswaart et al., 2011). Recent advances in brain–computer interface (BCI) technology offer a novel method that could extract the motor intention of patients executing MI to support active rehabilitation training. Related studies have shown promising results that MI-actuated BCI improves motor ability more than pure MI or sham BCI (Ramos-Murguialday et al., 2013; Ang et al., 2014; Pichiorri et al., 2015). However, this intervention still faces limitations in practical use (Mulder, 2007; Baniqued et al., 2021). First, BCI may not be easy for everyone due to the “BCI illiteracy” phenomenon or the limited training schedule in clinical environments (Blankertz et al., 2009; Horowitz et al., 2021). In addition, most stroke subjects show more difficulty executing MI tasks than healthy subjects because of brain impairment in motor-related areas (Mulder, 2007). Worse situations occur in severe patients because they can hardly perform effective MI or fall into fatigue quickly under effortful attempts. Recent studies found that action observation (AO) could also activate sensorimotor features, as in MI and motor execution tasks (Friesen et al., 2017; Hardwick et al., 2017). In addition, repeated AO could induce plasticity changes by activating the mirror neuron system (MNS) (Rizzolatti and Sinigaglia, 2010; Agosta et al., 2017). These inspired studies combined AO in the BCI system, where stronger event-related desynchronization (ERD) responses are found than in pure MI-BCI (Kondo et al., 2015; Ono et al., 2018; Nagai and Tanaka, 2019). However, most of these studies focused on healthy subjects, while related endeavors in the clinical rehabilitation of stroke subjects are still insufficient.

Another major concern in exploring novel interventions in chronic stroke is better evaluating the motor deficits and understanding the therapeutic-induced improvement during rehabilitation neurologically. On the one hand, the recovery in post-stroke motor rehabilitation is usually heterogeneous. Except for individual factors such as age, time since stroke, and related complications, a variety of neuro-clinical factors, such as the degree of brain lesion and neural status, would also affect the patient’s recovery (Riley et al., 2011; Chang et al., 2013; Feng et al., 2015; Kim and Winstein, 2017). On the other hand, chronic stroke recovery is more challenging with the decreasing plasticity of spontaneous recovery and depends more on intervention-induced plasticity (Cassidy and Cramer, 2017). The routinely used assessment of motor recovery is on clinical scales, which are semi-objective and limited in monitoring the underlying neural factors. Hence, recent studies have focused on finding neural biomarkers that could serve as an additional physiological approach to probe brain status and reflect the extent of post-stroke functional recovery (Kim and Winstein, 2017). Potential biomarkers have been found in physiological measuring tools such as Functional magnetic resonance imaging (fMRI) and magnetoencephalograms (MEG) (Várkuti et al., 2013; Kim and Winstein, 2017).

Compared with these tools, electroencephalography (EEG) offers another economical and widely available choice, making it a more practical approach in clinical environments for rehabilitation (Gerloff et al., 2006; Ang and Guan, 2016). In addition, the EEG is easy to implement in EEG-based BCI interventions. However, most related investigations of EEG markers focused on acute or subacute-stage patients, and studies concerned with chronic patients are still lacking (Foreman and Claassen, 2012; Assenza et al., 2017; Trujillo et al., 2017; Bentes et al., 2018). Notably, EEG oscillations in different bands themselves play roles in reflecting the physiological and pathological status of the neural systems. For example, the increasing low-frequency power (delta and theta bands) and decreasing high-frequency power (alpha and beta bands) are believed to reflect the severity of acute neurological deficits (Rabiller et al., 2015; Assenza et al., 2017). Apart from reflecting the motor status, the EEG features may also promote an understanding of varied recovery resulting from additional factors during rehabilitation. For instance, a previous study found that patients under different interventions have different EEG indicators (Mane et al., 2019). We infer that patients with varying degrees of recovery may also differ in EEG features after experiencing different neural processes in training. Overall, how these EEG oscillations would act in chronic stroke and whether related EEG features could reflect therapeutic-induced improvement in effective interventions remains to be determined.

To fill this gap, the present study aimed to explore whether brain oscillations in different EEG bands can reflect the motor status and recovery induced by novel BCI therapy in chronic stroke. Specifically, an AO-BCI robotic hand training intervention was studied in a clinical environment, and the motor scales were assessed before and after the training. The correlations between EEG band power and motor scales both before and after the intervention were analyzed to study their feasibility in reflecting motor status by EEG band power in chronic stroke patients. In addition, we presented the difference in EEG variation during an intervention on patients with and without effective recovery [whether the minimal clinically important difference (MCID) was reached] (van der Lee et al., 2001; Wagner et al., 2008). Moreover, we examined which EEG rhythm variations correlate with motor function improvement and their potential as markers in reflecting therapeutic-induced neuroplasticity changes and guiding rehabilitation intervention in chronic stroke patients.

## 2 Materials and methods

### 2.1 Subjects

All the subjects with chronic stroke were recruited via public information (the Hong Kong Stroke Association and hospitals) all over Hong Kong SAR, China. They had given their written, informed consent according to the Declaration of Helsinki. The Joint Chinese University of Hong Kong-New Territories East Cluster Clinical Research Ethics Committee (CUHK-NTEC CREC) approved the experimental protocol (agreement #2014.705-T). This study is also registered at www.clinicaltrials.gov with the study identifier NCT02323061. Subjects recruited in this study satisfied the following inclusion criteria: (1) had a unilateral ischemic brain injury or intracerebral hemorrhage at least 6 months after the onset of a single stroke without other diagnosed neurological deficits; (2) had sufficient cognition to follow simple instructions, as well as understand the content and purpose of the experiment assessed by Mini-Mental State Examination (MMSE>21) (Mowla and Zandi, 2006); (3) had moderate-to-severe motor disability at the paretic upper limb assessed by upper extremity Fugl–Meyer Assessment (FMA) (Fugl-Meyer et al., 1975) and Action Research Arm Test (ARAT) (van der Lee et al., 2002). In addition, subjects with the following reasons were excluded from the study: (1) cannot perform the training tasks for more than 30 min due to eye discomfort; (2) are not motivated to participate after being informed of the study details. This enrollment process resulted in 16 subjects being involved in the study. Eleven subjects finished the AO-driven BCI robotic hand training, and five subjects finished the sham-BCI training. The demographical and clinical characteristics of the recruited subjects are shown in Table 1. Age is expressed in years. Time since stroke (TSS) is expressed in years. ARAT: Action Research Arm Test ranged from 0 (most affected) to 57 (least affected); and FMA: Fugl-Meyer Assessment Scale, upper limb section ranged from 0 (most affected) to 66 (least affected). ARAT assessment forms for two stroke subjects (S6 and S7) were missing in the hospitals.

**Table 1:** Stroke subjects’ demographic data.

Subjects	Age(y)/Sex	TSS(y)	BCI Type	Affected hand	ARAT	FMA
S1	68/F	3	AO-BCI	Left	14	25
S2	65/M	8	AO-BCI	Right	10	22
S3	46/M	1	AO-BCI	Left	3	19
S4	48/F	1	AO-BCI	Left	8	36
S5	58/M	10	AO-BCI	Right	15	22
S6	65/M	4	AO-BCI	Right	\	25
S7	34/M	1	AO-BCI	Left	\	27
S8	59/M	11	AO-BCI	Left	28	24
S9	66/M	1	AO-BCI	Left	8	13
S10	46/M	1	AO-BCI	Left	16	17
S11	47/M	2	AO-BCI	Right	15	20
S12	48/F	3	Sham-BCI	Left	10	28
S13	45/M	2	Sham-BCI	Left	13	20
S14	46/M	1	Sham-BCI	Left	21	33
S15	58/F	3	Sham-BCI	Left	15	24
S16	56/M	5	Sham-BCI	Right	9	12
Mean	54.7	3.6	\	\	13.2	22.9
SD	10.6	3.2	\	\	5.9	6.2

### 2.2 Rehabilitation system and protocol

**Figure 1:**
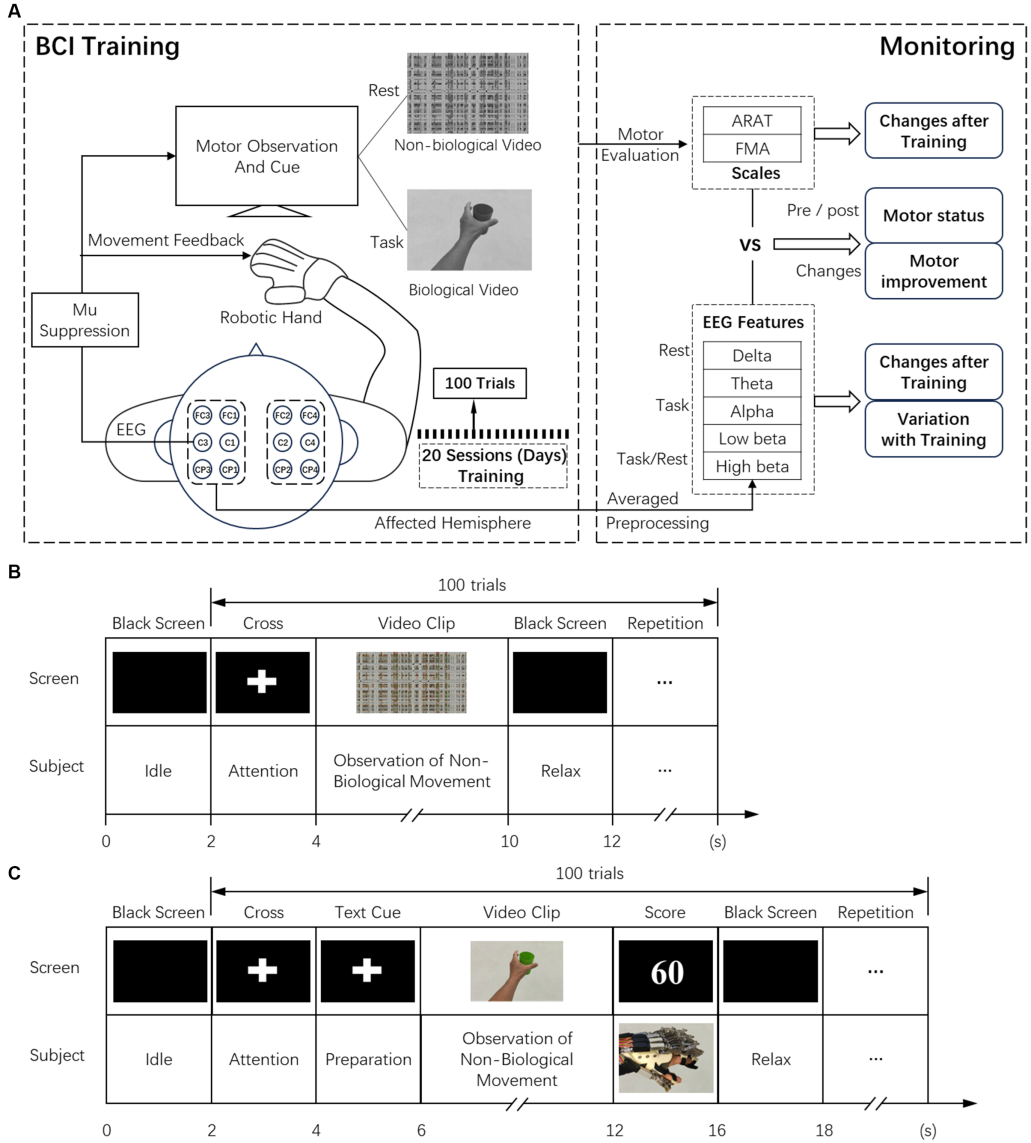
**(A)** Experimental setup of the BCI training and the analysis of offline data in biomarker analysis. **(B)** The timeline of recording resting state EEG while observation of non-biological movements. **(C)** The timing for BCI training while observation of biological movements.

All subjects received training of 20 sessions (days) within 5 ~ 7 weeks. ARAT and FMA clinical scales were applied pre-training, and immediately after the 20 sessions of training, EEGs were collected all over the training sessions. During the intervention, each subject was seated on a height-adjustable chair with their (1) right elbow positioned at 90 degrees abduction, (2) right elbow flexed 90 degrees, (3) right arm pronated such that the palm is directed medially, and (4) wrist positioned neutrally without any flexion/extension. An armrest supported and kept the subject’s arm in position. (5) The hand was put on the table comfortably. The experimental setup and training paradigm for BCI-based robotic hand intervention are shown in Figure 1 (Tong et al., 2013; Sun et al., 2017). In specific training sessions, subjects followed the instructions in training paradigms, each containing 100 repeated trials. Before the training task in each session, the baseline EEG of all subjects was measured in the resting state. Observation of biological and non-biological movement paradigms was used in the baseline measurement and training of patients in the AO-driven BCI intervention (Oberman et al., 2013; Frenkel-Toledo et al., 2014). The experimental conditions were as follows: (1) observation of biological movement: observing a video showing reaching and grasping a cup with the affected hand or a video showing releasing the cup. The video clip included 144 frames with 1920 × 1080 pixels in each frame. (2) Observation of non-biological movement: observing a video generated by decomposing the video clip of biological movement into frames (24 frames per second), and every frame was spatially scrambled (192*108 fragments in each frame), ensuring that the hand action could no longer be recognized. During the observation, no body movement or chewing was allowed. The timing of the experimental sequences and behavior tasks for observation of non-biological and biological movements are shown in Figures 1B,C. During the training stage of observing biological movements, 2 s of the dark screen were first displayed, followed by a white cross for 2 s. A text cue (hand grasp or open) was then displayed for 2 s. Then, a video clip with a duration of 6 s was shown. Subjects were asked to observe the actions and try to minimize eye-blinking. A robotic hand was activated as feedback based on the scores of mu suppression (Perry and Bentin, 2009; Tong et al., 2013; Sun et al., 2017). The trial ended with 2 s of dark screen. The experiment paradigm for observing non-biological movements in the resting stage was similar to biological movements except for different cues, and no feedback was provided. In addition to the patients experiencing sham-BCI, the timing of the paradigm was the same as in the AO-BCI training, except that the feedback was presented randomly. The presentation of the paradigm was controlled by the Psychophysics Toolbox 3.0 (Brainard and Vision, 1997).^1^

### 2.3 EEG acquisition and analysis

EEG signals were referenced to a unilateral earlobe, grounded at frontal position (Fpz), and sampled at 256 Hz using a g.USBamp (g.Tec Medical Engineering GmbH, Austria) system with 16 active electrodes (g.LADYbird). The active electrodes were composed of a sintered Ag/AgCl crown with a 2-pin safety connector. Compared with the passive electrodes, the active electrodes could improve the signal-to-noise ratio (SNR) to make the acquired EEG signals less affected by motion artifacts and electromagnetic interference. Electrodes were placed using the cap g.GAMMAcap (g.Tec Medical Engineering GmbH, Austria), thus allowing a fast placement. EEG signals were also online band-pass filtered from 2 to 60 Hz and notch-filtered between 48 and 52 Hz to remove artifacts and power line interference. All active electrodes were filled properly with conductive gel, and the active electrode system assured a transmission impedance of below 1 kOhm. According to the International 10–10 system, the electrodes were placed over the central area to obtain the neural activities related to the motor cortex. EEG data were analyzed using MATLAB (MathWorks, Natick, MA).

Many researchers believe that mu suppression is associated with the activation of MNS in human brains (Perry and Bentin, 2009; Bartur et al., 2015). In online analysis, mu suppression scores are calculated to provide feedback on training tasks. C3 or C4 was selected according to the subject’s ipsilesional side to compute the mu suppression. The value of the mu suppression score was equal to the negative difference in mu power between the observation of biological movement and non-biological movement, divided by the mu power during the observation of non-biological movement (the baseline) and multiplied by 100 (Oberman et al., 2008; Braadbaart et al., 2013; Sun et al., 2017).

In offline analysis, artifacts were rejected by visual inspection, and trials with artifacts were deserted (Kaya, 2019). Power spectra of artifact-free EEGs were computed using a Fast Fourier Transform at 0.5 Hz intervals (using a Hanning window). The mean spectral power (averaged of all artifact-free trials for each electrode) of each training session was calculated in the theta (4–8 Hz), mu (8–12 Hz), low-beta (16–20 Hz), and high-beta (20–24 Hz) frequency ranges (Pfurtscheller et al., 1998, 2002). Instead of restricting our analysis over C3 and C4 electrodes (Pineda, 2005; Oberman et al., 2013), we computed averaged power at more sites in the lesion brain that are related to motor function (left hemisphere: FC3, FC1, C3, C1, CP3, and CP1; right hemisphere: FC4, FC2, C4, C2, CP4, and CP2). These electrodes lie over the main areas related to motor function (including pre-motor, primary, and supplementary motor cortex) and related areas such as the somatosensory association cortex, which is involved in tactile sensation and perception of limb location. These are also engaged in identifying the postures and gestures of other people and also cover a major part of the mirror neuron system (Reed and Caselli, 1994; Carlson, 2012). Three kinds of EEG features in these bands are applied for further analysis: absolute band power in the resting stage, absolute band power in the task stage, and the ratio of task band power relative to resting-stage band power.

### 2.4 Statistical analysis

The main process is illustrated in Figure 1A. For proper estimation of the statistical significance with a small sample size, the non-parametric permutation testing method was employed (Nichols and Holmes, 2002; Philips et al., 2017; Trujillo et al., 2017). The two-tailed Spearman’s correlation was adopted for analyzing relationships between EEG power (in different bands) and clinical scales (ARAT and FMA), including pre-training, post-training, and variations after the intervention. We calculated the power using the QFAB Bioinformatics, ANZMTG Statistical Decision Tree, and Power Calculator, v1.0. Assuming a Spearman’s rank correlation coefficient of 0.7 ± 0.1 between the QEEG indices at T0 and motor outcome, the recommended sample size was 9–19 subjects to achieve a statistical power of 80% with a significance level of 0.05. Thus, the sample size of 16 in this study was sufficient. The Bonferroni correction was used to adjust the alpha, which provides a conservative method to address the type I error in multiple comparisons. An adjusted alpha value of 0.01 was used to correct for analysis across five frequency bands.

The comparisons between the clinical scales before and after intervention were made using the paired Wilcoxon signed-rank test. The unpaired Wilcoxon signed-rank test was also used to compare the difference in power variation in 20 sessions between subjects with (reaching MCID, ARAT: 5.7 points, FMA: 5.2 points) and without (not reaching MCID) effective recovery. All statistical work was performed using SPSS 19 (SPSS Inc., Chicago, Illinois, USA).

## 3 Results

### 3.1 Clinical improvements

The clinical scale before and after training is shown in Supplementary Table S1. Significant changes in ARAT and FMA after intervention were found compared with scales before training (△ARAT, 6.1 ± 6.8, *p* = 0.017; △FMA, 3.7 ± 4.4, *p* = 0.005). In addition, 82% of these patients improved more than the MCID in ARAT or FMA scores after the AO-BCI intervention.

### 3.2 EEG power and motor status

**Figure 2:**
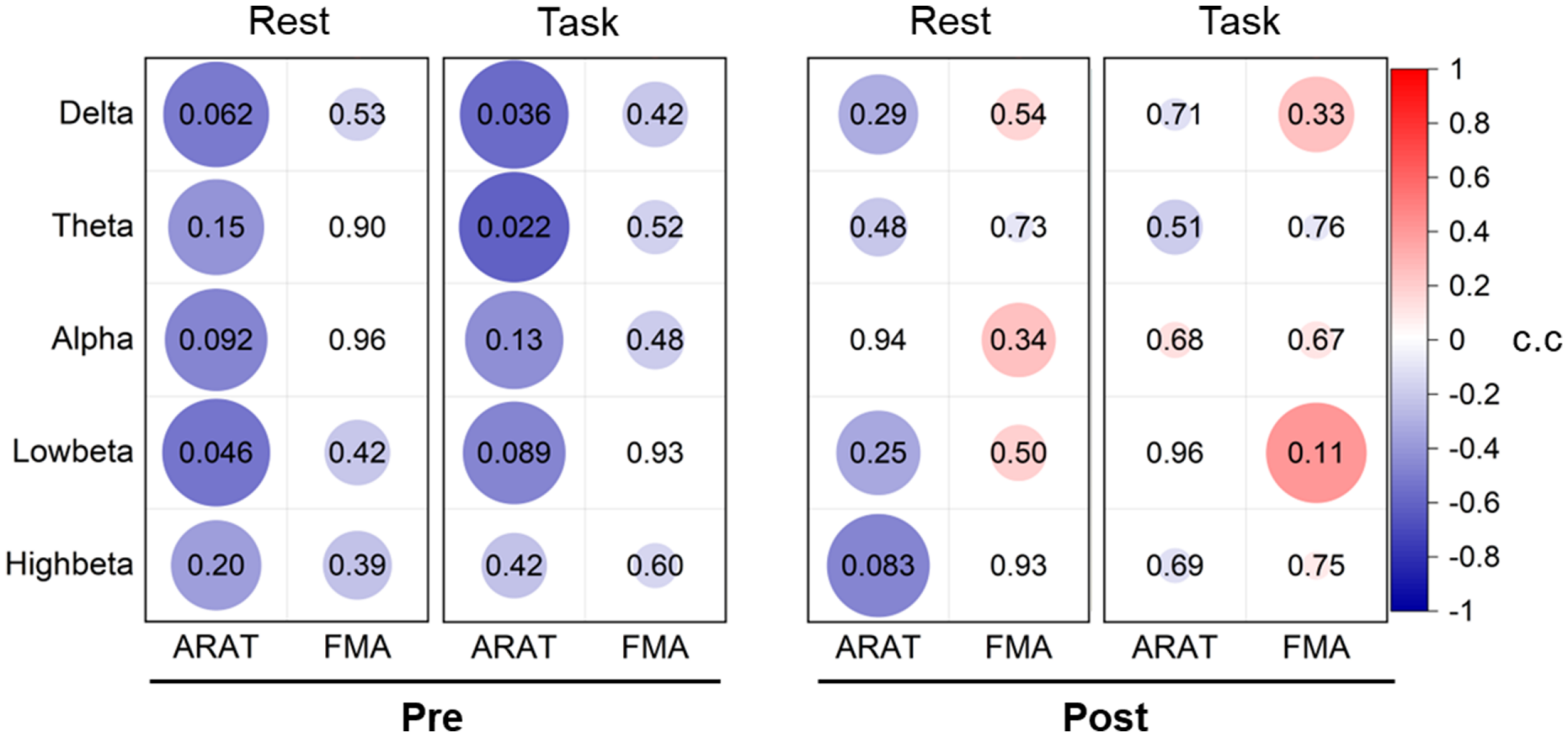
Correlation analysis between averaged contralateral EEG power and clinical scales pre-training and post-training. The values of the correlation coefficient (c.c) are presented in the color of circles. The value of *p*s are presented with the number in circles and related to the size of the circles (larger size corresponds to the lower value of *p*).

To investigate whether the EEG power feature could reflect motor status, we analyzed the correlation between the contralateral average EEG power and the clinical scales (Supplementary Tables S2, S3). However, no significant results were found both in pre-training and post-training data, as presented in Figure 2, which signifies that EEG power is not an effective indicator for monitoring motor status. Nonetheless, we found negative correlations between EEG power in all bands and motor status before training both during the rest and the task stages, especially in delta, theta, and low-beta frequencies (rest: low-beta c.c = −0.54, *p* = 0.046; task: delta c.c = −0.56, *p* = 0.036, and theta c.c = −0.61, *p* = 0.022) with ARAT. After training, these correlation relationships varied and tended to weaken negative or even positive correlations.

### 3.3 EEG variations

Comparing the averaged EEG power of all patients before and after the intervention, we did not find any significant differences in any frequency bands (as presented in Supplementary Table S4). However, in patients with effective recovery (reaching the MCID in ARAT or FMA in 9 subjects), we found improvements with significant levels in delta band power (task and task/rest, *p* < 0.01), as presented in Supplementary Table S5.

**Figure 3:**
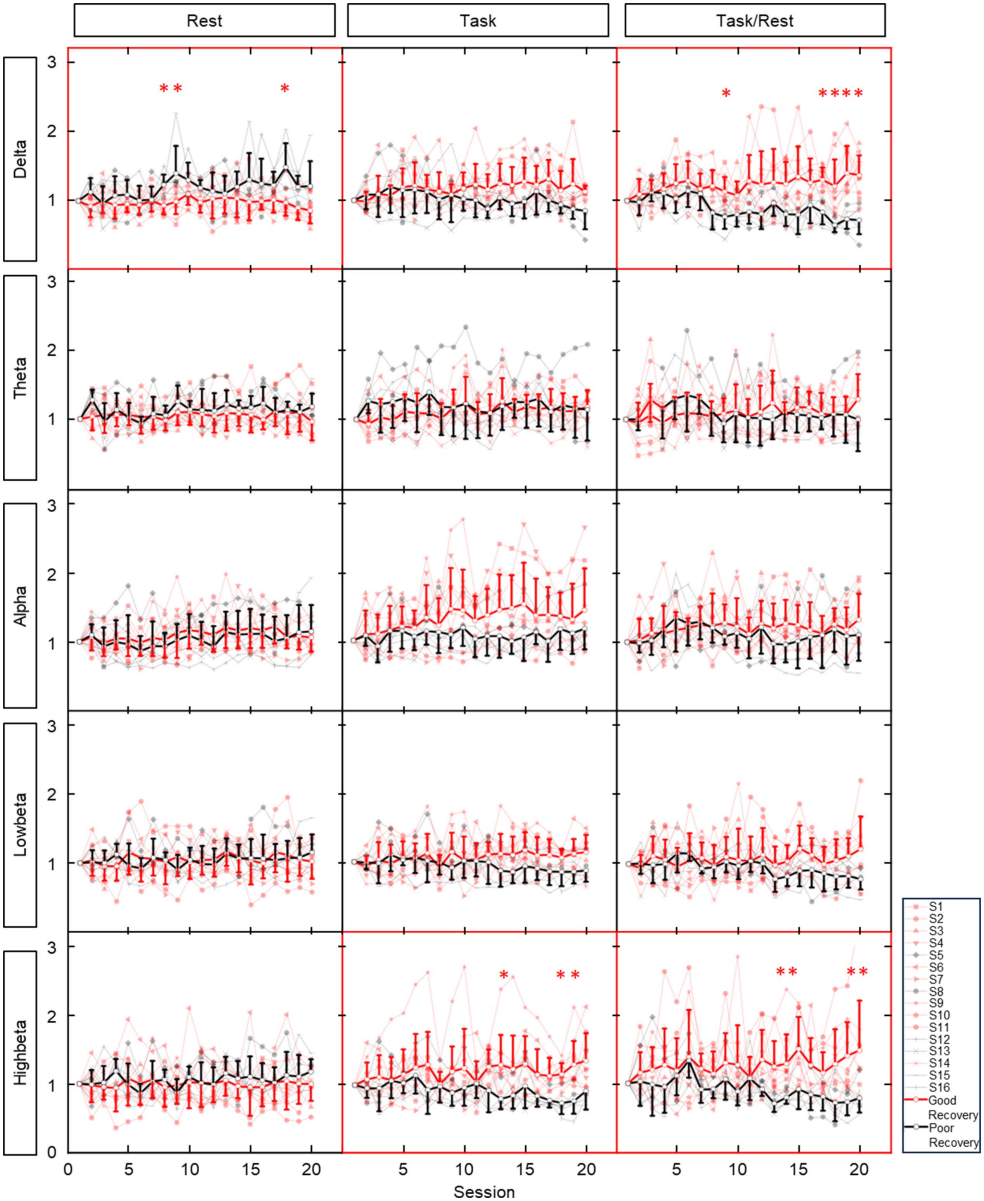
Average ipsilesional EEG power variation (relative to the first session) during 20 sessions of training. Rows show EEG in different bands. Columns show EEG in different states. Red lines and dots: patients with good recovery (reaching the MCID level). Black lines and dots: patients with poor recovery (not reaching the MCID level). Bold and dark lines: averaged data from patients with and without effective recovery. * indicates the significant (*p* < 0.01) result in corresponding training session. ** represent significant results in two consecutive sessions.

The averaged contralateral EEG power variation (relative to the first session) during 20 sessions of training is presented in Figure 3. Considerable power differences were found in resting-state delta band EEG between patients with or without effective recovery, in which patients with better recovery showed lower delta power with training. In task EEG, patients with effective recovery showed higher power in the high-beta bands (*p* < 0.01 in three sessions). The task/rest power presented the results with both higher power in the delta (*p* < 0.01 in five sessions) and high-beta (*p* < 0.01 in four sessions) bands.

### 3.4 EEG variation and motor recovery

**Figure 4:**
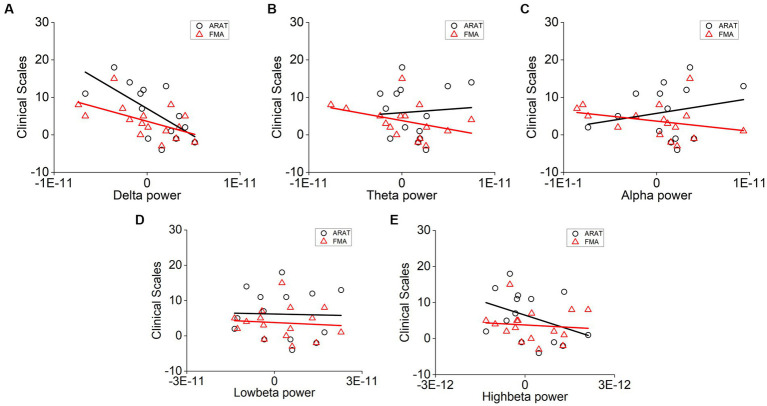
Correlation analysis of averaged **(A)** delta, **(B)** theta, **(C)** alpha, **(D)** low-beta, and **(E)** high-beta power variation in the affected hemisphere during rest and clinical scale variation after 20 sessions of training. Each point denotes data from one subject. Correlation analyses: two-tailed Spearman’s correlation coefficient.

**Figure 5:**
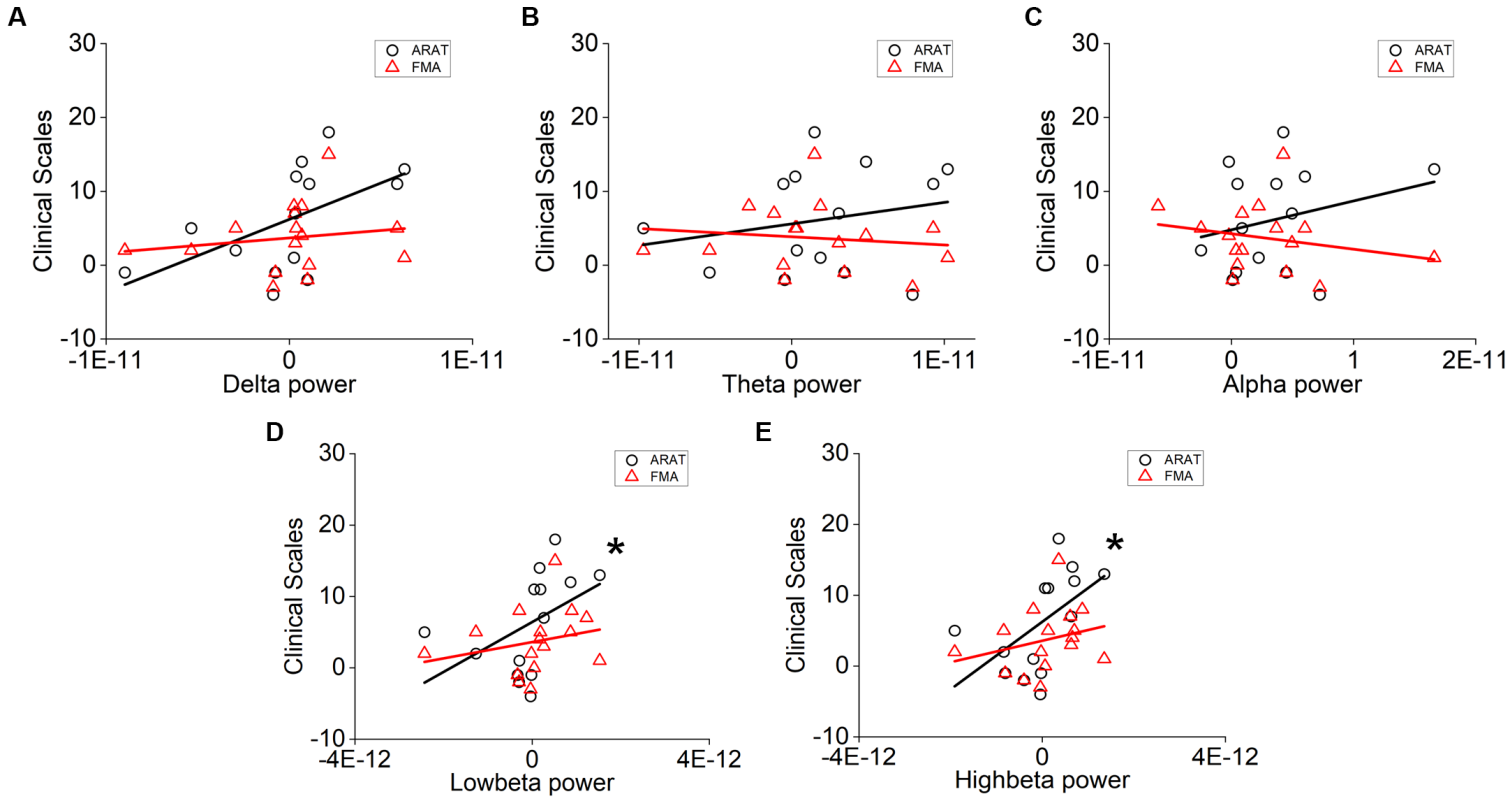
Correlation analysis of averaged **(A)** delta, **(B)** theta, **(C)** alpha, **(D)** low-beta, and **(E)** high-beta power variation in the affected hemisphere during task and clinical scale variation after 20 sessions of training. Correlation analyses: two-tailed Spearman’s correlation coefficient (Significance: *p < 0.01).

**Figure 6:**
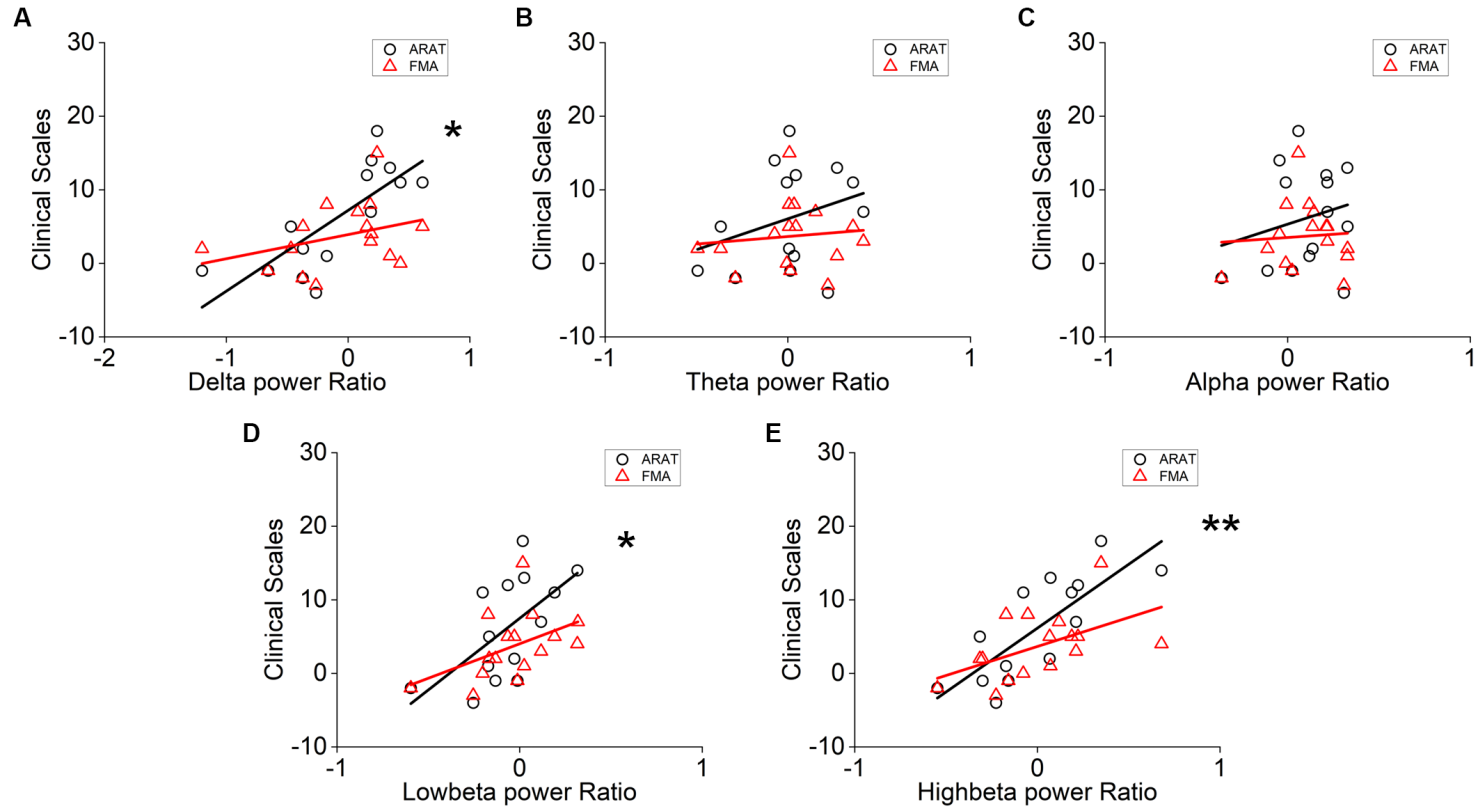
Correlation analysis of averaged **(A)** delta, **(B)** theta, **(C)** alpha, **(D)** low-beta, and **(E)** high-beta task/rest power ratio variation in the affected hemisphere and clinical scale variation after 20 sessions of training. Correlation analyses: two-tailed Spearman’s correlation coefficient. (Significance: *p < 0.01, **p < 0.001).

**Figure 7:**
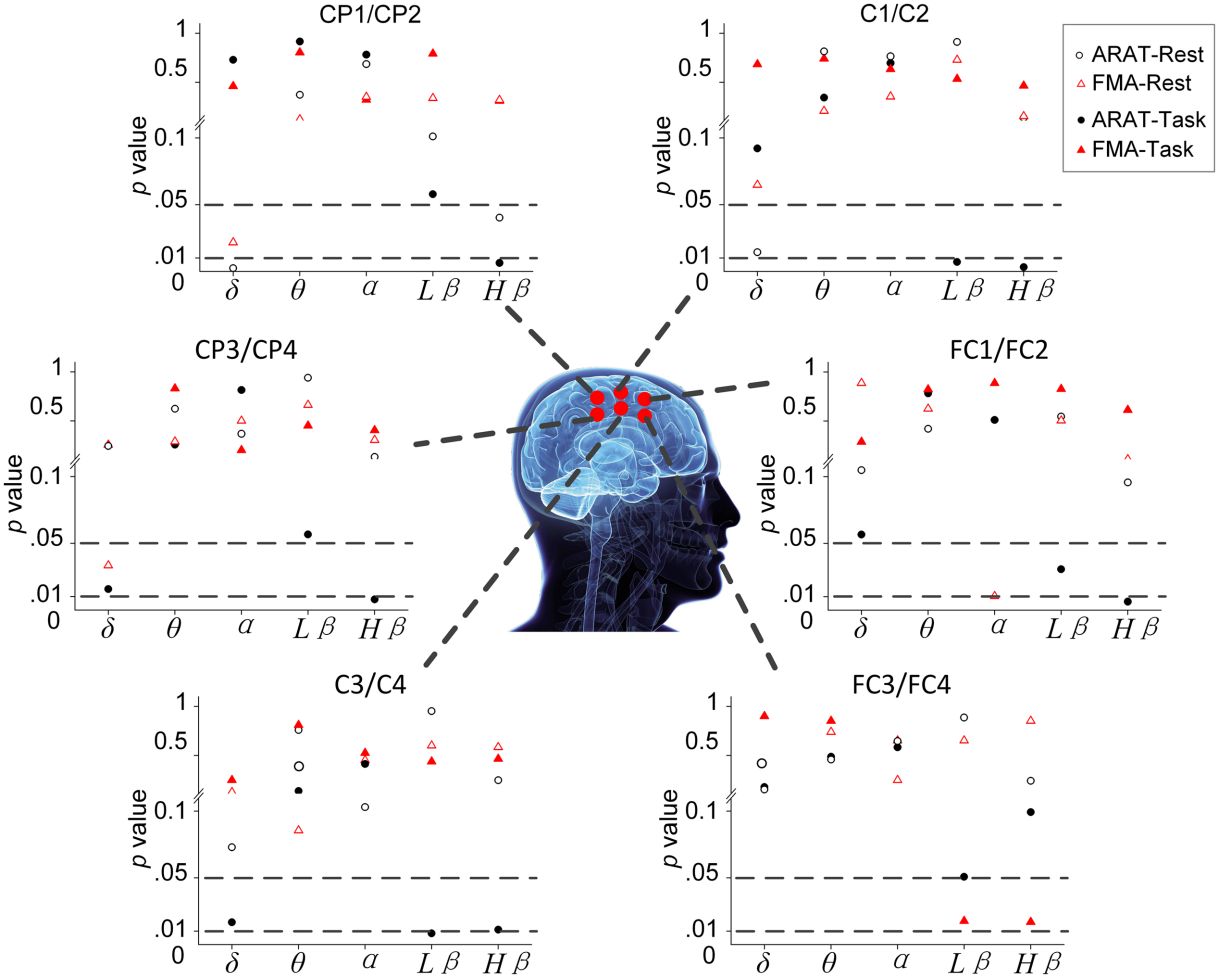
Significance of correlation between clinical improvements and variation of EEG power during rest and task in different electrodes.

The correlation between averaged EEG variation after training in the affected hemisphere and motor improvements is presented in Table 2. Specifically, no significant negative correlations were found between band power changes during the resting stage and clinical improvements in ARAT or FMA, as shown in Figure 4. In task EEG (Figure 5), significant positive correlations between clinical improvements in ARAT and absolute power changes in the low-beta (c.c = 0.71, *p* = 0.005) and high-beta (c.c = 0.71, *p* = 0.004) bands were found. The most significant correlations with clinical improvements were found in the task ratio EEG power variation, in which positive correlations were found in the delta (c.c = −0.738, *p* = 0.003), low-beta (c.c = 0.67, *p* = 0.009), and high-beta (c.c = 0.839, *p* = 0.0002) bands (Figure 6). We further analyzed the correlation between clinical improvements and variations in EEG power in different electrodes (Figure 7). For delta power, significant results (*p* < 0.01) were found in the electrode of CP1/CP2 (ARAT) during rest. For beta power, significant results were found in almost all involved electrodes (C1/C2, C3/C4, CP1/CP2, CP3/CP4, and FC1/FC2) during the task. To prove the robustness of the observed effects, we further illustrated the variation of the averaged correlation coefficient and value of *p* (ΔEEG power vs. ARAT improvements) as a function of the number of subjects and trials in Supplementary Figures S2, S3. These correlation coefficients and *p*-values with significant results above were presented to be convergent with the increasing number of subjects and trials.

**Table 2:** Correlation analysis of EEG variation and clinical scales improvements after training.

EEG state		Delta		Theta		Alpha		Lowbeta		Highbeta	
		ARAT	FMA	ARAT	FMA	ARAT	FMA	ARAT	FMA	ARAT	FMA
Rest	C.C	−0.652	−0.538	−0.015	−0.448	0.099	−0.430	−0.159	−0.171	−0.407	−0.106
	*p*	0.012	0.032	0.958	0.082	0.736	0.097	0.588	0.526	0.148	0.695
Task	C.C	0.637	0.134	0.200	−0.162	0.119	−0.216	**0.707**	0.406	**0.714**	0.331
	*p*	0.014	0.620	0.492	0.548	0.685	0.422	**0.005**	0.118	**0.004**	0.211
Task ratio	C.C	**0.738**	0.309	0.189	0.136	0.137	−0.001	**0.665**	0.507	**0.839**	0.541
	*p*	**0.003**	0.245	0.517	0.616	0.642	0.996	**0.009**	0.045	**0.000**	0.031

C.C., Correlation coefficients; *p*, *p* value. Significant results (*p* < 0.01) are bolded.

## 4 Discussion

### 4.1 EEG power and motor status

Previous studies that used EEG to reflect neurological deficits focused on acute rather than chronic stroke (Assenza et al., 2013; Wu et al., 2016; Assenza et al., 2017). These studies found that higher delta and lower beta waves signify more severe motor deficits post-stroke (Assenza et al., 2009; Dubovik et al., 2012; Finnigan and van Putten, 2013; Assenza et al., 2017). In this study, we explored the utility of potential EEG biomarkers in the chronic stage and whether the well-established role of EEG band power in reflecting motor status in early stroke could also be applicable in chronic stroke. However, we found no significant correlation (*p* < 0.01) between band power and motor scales both before and after the training, which may signify that EEG band power is not a reliable indicator of motor status in the chronic stage. Despite that, the correlation results also provide information to look into chronic stroke motor rehabilitation.

Specifically, in pre-training results, the ipsilesional delta waves (during rest and task stages) in the affected hemisphere and motor functions (ARAT and FMA) presented a negative correlation. This result also aligns with previous evidence in chronic stroke that increased low-frequency power is associated with brain lesions as measured by infarct volume (Cassidy et al., 2020; Barone and Rossiter, 2021). In the acute stage, these pathological changes would immediately affect motor function, thus also presenting a negative correlation with functional scales. The same negative correlation result in our study may signify that pathological changes last to the chronic stage (Barone and Rossiter, 2021). Currently, to the best of our knowledge, few groups have explored the correlation between EEG delta power and motor status in the chronic stage. Several recent studies investigated the quantitative EEG indicators (the ratio between EEG power in different frequency bands) instead of the absolute EEG power, as in our study (Finnigan and van Putten, 2013; Assenza et al., 2017). Although we got consistent results between low-frequency activity and motor status, no directly significant correlations were found either. For the results after the intervention, the correlation between delta power and motor scales changed in an inverse direction (weaker negative or positive correlation) compared with the pre-training results. Hence, these results may signify that, except for the initial brain impairments, other factors in later rehabilitation may also influence the EEG power in chronic stroke, thus making these correlation results insignificant or tending to be weaker.

Lower beta activity in acute stroke is usually associated with worse behavioral function (Assenza et al., 2009; Dubovik et al., 2012; Assenza et al., 2017). However, the pre-training result of our study indicates that this finding may not be applicable to patients in the chronic stage. On the contrary, we found excessive ipsilesional beta power during rest reflects worse motor function. Two roles of beta power have been found, which reflect the stroke-induced cortical deficits (decreased beta activity is generated for the death of brain cells in the affected area) or the compensatory changes in the motor deficit (increased beta power for the higher effort required for motor tasks) (Pogosyan et al., 2009; Foreman and Claassen, 2012; Rossiter et al., 2014; Rabiller et al., 2015; Guerra et al., 2019). Previous studies in acute stroke support the former, while the result of higher beta power indicating worse motor function in our study is consistent with the latter. Only one directly related study in chronic stroke was found, and it supports our result that resting-stage beta band power in the affected hemisphere is associated with poor motor function (Thibaut et al., 2017). For the post-training results on the beta band, we found a similar variation as that in the delta band, in which weaker negative or inversely positive correlations were found compared with the pre-training results.

### 4.2 EEG variations and motor recovery

Considering the inconsistent correlations (band power vs. motor status) in the pre-training and post-training data, we investigated the intervention-induced changes in these EEG band powers (Supplementary Table S4). However, we did not find significant differences (*p* < 0.01) between pre-training and post-training EEG power. This result is consistent with one previous study and signifies that EEG power could not directly reflect the intervention mechanism during chronic rehabilitation (Trujillo et al., 2017). However, EEG changes in patients with effective recovery presented significant changes in delta band power (task and task/rest), as presented in Supplementary Table S5. Hence, we suspect that the different levels of recovery in training may also influence the EEG power for the underlying different neural activities, and we think that they should be considered together.

Comparisons between patients with and without effective recovery further verified that, during chronic stroke rehabilitation, the variation of EEG power is also influenced by the degree of recovery. As shown in Figure 3, patients with different degrees of recovery presented different trends in EEG power variations during training, and significant differences were found in the delta, low-beta, and high-beta bands. The significant correlation results between EEG power changes (delta, low-beta, and high-beta) and motor recovery also provided consistent supporting evidence.

#### 4.2.1 Delta band

Specifically, subjects with better motor improvement showed higher delta power (task and task/rest) increases. This observation is consistent with previous literature, in which Assenza et al. performed intermittent theta burst stimulation (iTBS) on healthy subjects and found that iTBS-induced plasticity can increase both motor-evoked potential (MEP) amplitude and delta power significantly (Assenza et al., 2015; Assenza and Di Lazzaro, 2015). The authors demonstrated that delta waves during wakefulness, as well as those found during sleep in animal studies, are a sign of brain plasticity (Huber et al., 2004; Tononi and Cirelli, 2012). Hence, increased delta power in the chronic stage may indicate intervention-induced plasticity change, therefore resulting in motor improvements after training. EEG variation during sessions in our study also showed consistent results, in which patients with better recovery (with MCID of clinical scales) presented higher delta power than patients with worse recovery (without MCID of clinical scales) over 20 sessions. In contrast, delta power variation at rest showed a negative correlation with motor improvement. This result may be interpreted as an increased demand for brain metabolism activity after effective training (Nagata, 1995; Finnigan and van Putten, 2013). Further investigation demonstrated that these recovery-related changes at rest mainly cover the region of the parietal lobe (CP1/CP2), as demonstrated in Figure 7 (Koessler et al., 2009; Scrivener and Reader, 2022). Considering the physiological role of this area, this result may signify that increased brain metabolic activity is used for motor learning, somatosensory integration, and activation of the MNS even after training (Trobe, 2010). In addition, we found that delta power ratio (task/rest) variation (c.c = −0.637, *p* = 0.014) showed a stronger positive correlation than delta power variation during the motor-related task (c.c = −0.738, *p* = 0.003). It seems that opposite results of rest and task beta power makes the variation of task/rest delta power a better marker of intervention-induced recovery.

#### 4.2.2 Beta band

Pre- and post-intervention results showed that both low-beta and high-beta power variations positively correlate with intervention-induced motor improvements. Significant results were found in the task beta power and task/rest beta power ratios, whereas non-significant results were found in the resting-state beta power. Results of EEG variation during 20 sessions of training also presented that those patients with better recovery (reaching the MCID) had increased beta power (especially in the high-beta bands during task and task power ratio). In other words, the increased task-related beta power is a positive indicator of motor recovery. Previous studies have shown that beta oscillation plays a role in motor learning (Espenhahn et al., 2019; Barone and Rossiter, 2021). Thus, in our study, patients with increased beta power may undergo a more effective learning process and hence reach better improvements. In addition, the beta wave has also been associated with gamma-aminobutyric acid (GABA) levels. Both human and animal studies showed that decreased GABA levels would cause increased beta power (Feshchenko et al., 1997; Van Lier et al., 2004). Since GABA plasticity plays an important role in stroke recovery, our results suggest that increased beta power during training may indicate induced GABA plasticity changes (Paik and Yang, 2014; Blicher et al., 2015). Consistent evidence is also found in other studies. For example, non-invasive cortical stimulation that enhances beta oscillations can promote motor learning and induce long-term plasticity in the motor cortex (Reis et al., 2009; Pollok et al., 2015). For the study of intervention, Penolazzi et al. (2010) found that beta waves in the EEG could be a marker of brain plasticity in phonological training for dyslexic children (Penolazzi et al., 2010). Collectively, our study demonstrated, for the first time, that the beta activity variation could be an indicator of motor relearning and recovery-related plasticity changes induced in a 20-session BCI motor therapy program for chronic stroke. We further investigated which electrodes would present these significant correlations between EEG features and improvements (Figure 7). Almost all involved electrodes (C1/C2, C3/C4, CP1/CP2, CP3/CP4, and FC1/FC2) in beta power presented significant results, which signify that during the task, the motor learning process and the plasticity changes reflected by beta activity occurred in extensive areas related to motor function (Trobe, 2010).

### 4.3 AO-BCI intervention and clinical improvements

Motor recovery in chronic stroke is challenging due to the decreasing plasticity of spontaneous recovery (Cassidy and Cramer, 2017). In this study, the AO-driven intervention significantly improved motor function in chronic stroke (FMA and ARAT were 5 ± 7.1, *p* < 0.01, and 7.9 ± 5.13 points, *p* < 0.05), which is presented to be an effective complementary therapy to other BCI interventions for motor rehabilitation (Pichiorri et al., 2015). This improvement is comparable with that in recent BCI-based motor rehabilitation studies (Mane et al., 2020). Besides, compared with the MI-BCI therapy, the AO-driven therapy relies less on patients’ active ability as it training by action observation with the assistance of the mirror neuron system (Rizzolatti and Sinigaglia, 2010; Ramos-Murguialday et al., 2013; Agosta et al., 2017). This intervention offers another accessible choice for patients with difficulty executing motor imagery due to severe injury or the “BCI illiteracy” phenomenon. More importantly, we found that this motor recovery is correlated with the EEG power variation in the delta and beta bands, which was associated with motor learning and plasticity change (Assenza et al., 2017; Guerra et al., 2019). These findings support the broader application of the AO-driven robotic hand intervention in future clinical rehabilitation to promote chronic-stage motor improvements and induce recovery-related plasticity change in the brain.

In this chronic stroke rehabilitation study, EEG power and EEG power variation showed different trends in indicating better status or recovery. Unlike previous results found in acute stroke, in which higher delta and lower beta power are directly related to severe brain injury, our results signify that these EEG powers in chronic stages may underline different mechanisms (Assenza et al., 2013; Finnigan and van Putten, 2013; Rabiller et al., 2015; Assenza et al., 2017). The pre-training EEG is related to the status of injury or function, while the EEG during training is a marker of plasticity change. Previous studies have found that spontaneous mechanisms mainly contribute to recovery in the acute stage, while recovery in the chronic stage is more dependent on therapeutic-induced mechanisms due to endogenous repair-related events becoming stable after 3 months post-stroke (Cramer et al., 2007; Overman and Carmichael, 2014; Bernhardt et al., 2017; Cassidy and Cramer, 2017). However, future cohort studies are needed to investigate these variations dynamically by following patients from the acute to the chronic stage.

The ARAT and FMA scales consistently correlated with delta and beta band oscillations. However, significant findings were more prevalent in the ARAT scale than the FMA scale, and a greater percentage of patients showed improvement beyond the minimal clinically important difference (MCID) in ARAT (50%) compared to FMA (25%). Considering that we specifically aimed at the hand training intervention and ARAT is more elaborate in reflecting hand function than upper extremity FMA, this may explain why ARAT showed more significant results in our study (Fugl-Meyer et al., 1975; van der Lee et al., 2002). In addition, previous studies have found that for intervention in chronic stroke, ARAT is more responsive than upper-extremity FMA (van der Lee et al., 2001). Here, we provide neurophysiology evidence supporting ARAT as the clinical evaluation of upper-extremity motor function in chronic stroke. Notably, De Weerdt and Harrison suggested that FMA reflects more at the impairment level, while ARAT reflects more at the disability level (De Weerdt and Harrison, 1985). This is consistent with the delta and beta power variations in effective recovery as discussed above and may signify that the AO-based intervention would contribute to recovery at the disability level more than the impairment level.

### 4.4 Prospects and limitations

We explored the electrophysiological biomarkers in chronic stroke during the intervention and found that EEG power (in the delta and beta bands) effectively reflects motor status and intervention-induced plasticity change. These results presented a useful electrophysiological tool for evaluating motor function and provided new insight into exploring neurophysiological variation during effective rehabilitation. However, future studies providing further verification on a larger scale are still needed to provide more substantial clinical evidence. Considering current research in the chronic stage is still scarce and small-sampled, studies employing multiple evaluation approaches may also help by providing multidimensional cross-evidence. For example, in addition to electrophysiological information, combining fMRI or rTMS with EEG would give a deeper understanding of these plasticity changes with extra spatial detail from hemodynamics and motor pathway physiology (Cassidy and Cramer, 2017).

## Data availability statement

The raw data supporting the conclusions of this article will be made available by the authors, without undue reservation.

## Ethics statement

The studies involving humans were approved by Joint Chinese University of Hong Kong-New Territories East Cluster Clinical Research Ethics Committee. The studies were conducted in accordance with the local legislation and institutional requirements. Written informed consent for participation in this study was provided by the participants’ legal guardians/next of kin. Written informed consent was obtained from the individual(s) for the publication of any potentially identifiable images or data included in this article.

## Author contributions

ZY and PX worked together to complete the manuscript. JW and RT contributed to the conception and design of the study, and carried out the experiments. ZY and JW carried out the data analysis. All authors contributed to the manuscript revision, and read and approved the submitted version.

## Footnotes

^1^ http://psychtoolbox.org/
