# Supplementary material for: Brain oscillations in reflecting motor status and recovery induced by action observation-driven robotic hand intervention in chronic stroke
Source: Front Neurosci. 2023 Dec 11;17:1241772. doi: 10.3389/fnins.2023.1241772 (PMC10749335; doi:10.3389/fnins.2023.1241772)
Supplement: Supplementary file 1 [file Data_Sheet_1.PDF]

Supplementary Material

Brain oscillations reflect motor status and recovery induced by Action-observation-driven robotic hand intervention in chronic stroke

Zan Yue, Peng Xiao, Jing Wang*, Raymond Kai-yu Tong*

*** Correspondence:** Jing Wang: wangpele@gmail.com;

Raymond Kai-yu Tong: kytong@cuhk.edu.hk

1 Supplementary Tables

1.1 Clinical Improvements

Subjects	ARAT			FMA		
	Pre	Post	Difference	Pre	Post	Difference
S1	14	27	13	25	26	1
S2	10	21	11	22	27	5
S3	3	21	18	19	34	15
S4	8	20	12	36	41	5
S5	15	14	-1	22	24	2
S6	\	\	\	25	33	8
S7	\	\	\	27	34	7
S8	28	24	-4	24	21	-3
S9	8	15	7	13	16	3
S10	16	17	1	17	25	8
S11	15	29	14	20	24	4
S12	10	12	2	28	33	5
S13	13	12	-1	20	19	-1
S14	21	32	11	33	33	0
S15	15	13	-2	24	22	-2
S16	9	14	5	12	15	2
Mean (AO-BCI)	13.00	20.89	7.89	22.73	27.73	5.00
SD (AO-BCI)	6.68	4.84	7.16	5.69	6.77	4.43
Mean (Shame-BCI)	13.60	16.60	3.00	23.40	24.40	0.80
SD (Shame -BCI)	4.27	7.74	4.69	7.14	7.36	2.48
Mean (all)	13.21	19.36	6.14	22.94	26.69	3.69
SD (all)	5.94	6.38	6.80	6.19	7.13	4.38

Table S1. Clinical outcome measures at pre- and post- assessments. For FMA-UE and ARAT, the minimally clinical important difference (MCID) of clinical scales is 5.2 points and 5.7 points for chronic stroke patients. Subjects reaching MCID on ARAT or FMA are reprented in red color.

EEG state		Delta		Theta		Alpha		Lowbeta		Highbeta	
		ARAT	FMA	ARAT	FMA	ARAT	FMA	ARAT	FMA	ARAT	FMA
**Rest**	C.C	-0.511	-0.171	-0.394	-0.033	-0.464	0.023	-0.540	-0.218	-0.365	-0.233
	p	0.062	0.526	0.163	0.903	0.095	0.933	0.046	0.417	0.199	0.385
**Task**	C.C	-0.562	-0.217	-0.606	-0.174	-0.420	-0.19	-0.462	-0.028	-0.232	-0.142
	p	0.036	0.42	0.022	0.519	0.135	0.480	0.096	0.918	0.424	0.601
**Task Ratio**	C.C	0.049	0.013	0.192	0.032	0.155	-0.094	0.261	0.277	0.069	0.118
	p	0.869	0.961	0.51	0.905	0.597	0.728	0.367	0.298	0.816	0.663

C.C.: Correlation coefficients, *p*: *p* value with Bonferroni correction (Significance: $*p<0.01$)

Table S2. Correlation analysis of EEG and clinical scales before training（corrected with Bonferroni correction）. No significant correlation could be found.

EEG state		Delta		Theta		Alpha		Lowbeta		Highbeta	
		ARAT	FMA	ARAT	FMA	ARAT	FMA	ARAT	FMA	ARAT	FMA
**Rest**	C.C	-.082	.304	-.152	.147	.049	.300	-.148	.117	-.265	-.055
	p	.782	.252	.603	.587	.869	.259	.614	.667	.361	.841
**Task**	C.C	-.287	.055	-.355	-.043	-.108	.055	-.253	.194	-.344	.003
	p	.320	.841	.213	.875	.713	.841	.383	.471	.228	.991
**Task Ratio**	C.C	-.501	-.453	-.088	-.180	-.284	-.233	-.101	.084	-.357	-.069
	p	.068	.078	.764	.504	.324	.384	.730	.757	.210	.798

Table S3. Correlation analysis of EEG and clinical scales after training（corrected with Bonferroni correction）. No significant correlation could be found.

EEG state		Delta		Theta		Alpha		Lowbeta		Highbeta	
		Pre	Post	Pre	Post	Pre	Post	Pre	Post	Pre	Post
**Rest**	Mean	1.52E-11	1.40E-11	2.26E-11	2.30E-11	2.06E-11	2.05E-11	4.67E-12	4.87E-12	3.67E-12	3.92E-12
	Std	1.00E-11	7.88E-12	1.38E-11	1.46E-11	1.17E-11	9.70E-12	2.08E-12	2.44E-12	1.51E-12	2.03E-12
	p	0.191		0.642		0.877		0.569		0.278	
**Task**	Mean	1.69E-11	1.72E-11	1.90E-11	2.23E-11	1.42E-11	1.79E-11	4.04E-12	4.11E-12	2.89E-12	3.26E-12
	Std	1.02E-11	1.12E-11	1.03E-11	1.22E-11	6.58E-12	7.65E-12	1.83E-12	1.83E-12	1.26E-12	1.55E-12
	p	0.408		0.024		0.063		0.717		0.326	
**Task Ratio**	Mean	1.23	1.27	0.97	1.06	0.82	0.96	0.90	0.89	0.82	0.91
	Std	0.46	0.51	0.33	0.34	0.35	0.36	0.21	0.29	0.26	0.36
	p	0.569		0.501		0.044		0.717		0.642	

Table S4. EEG power of all patients (n=16) recorded at the first session(pre-training) and the last session (post-training). Differences are analyzed by Wilcoxon signed-rank test with Bonferroni correction. No significant correlation could be found.

EEG state		Delta		Theta		Alpha		Lowbeta		Highbeta	
		Pre	Post	Pre	Post	Pre	Post	Pre	Post	Pre	Post
**Rest**	Mean	1.73E-11	1.43E-11	2.17E-11	2.03E-11	2.27E-11	2.29E-11	5.11E-12	5.27E-12	3.98E-12	4.16E-12
	Std	1.13E-11	8.77E-12	1.26E-11	1.13E-11	1.27E-11	1.06E-11	2.07E-12	2.82E-12	1.61E-12	2.43E-12
	p	0.022		0.386		0.959		0.721		0.878	
**Task**	Mean	1.60E-11	1.83E-11	1.72E-11	2.13E-11	1.42E-11	1.89E-11	4.06E-12	4.66E-12	2.71E-12	3.62E-12
	Std	1.03E-11	1.22E-11	7.82E-12	1.13E-11	5.87E-12	6.90E-12	1.31E-12	1.80E-12	1.02E-12	1.68E-12
	p	**0.009**		0.022		0.114		0.050		0.028	
**Task Ratio**	Mean	1.02	1.39	0.92	1.16	0.76	0.97	0.84	0.97	0.71	1.01
	Std	0.35	0.58	0.27	0.38	0.36	0.41	0.21	0.32	0.21	0.40
	p	**0.009**		0.103		0.011		0.308		0.047	

Table S5. EEG power of patients with effective recovery (n=10, reaching MCID in ARAT or FMA) recorded at the first session(pre-training) and the last session (post-training). Differences are analyzed by Wilcoxon signed-rank test with Bonferroni correction (α =0.01). Significant values are bolded.

2 Supplementary Figures

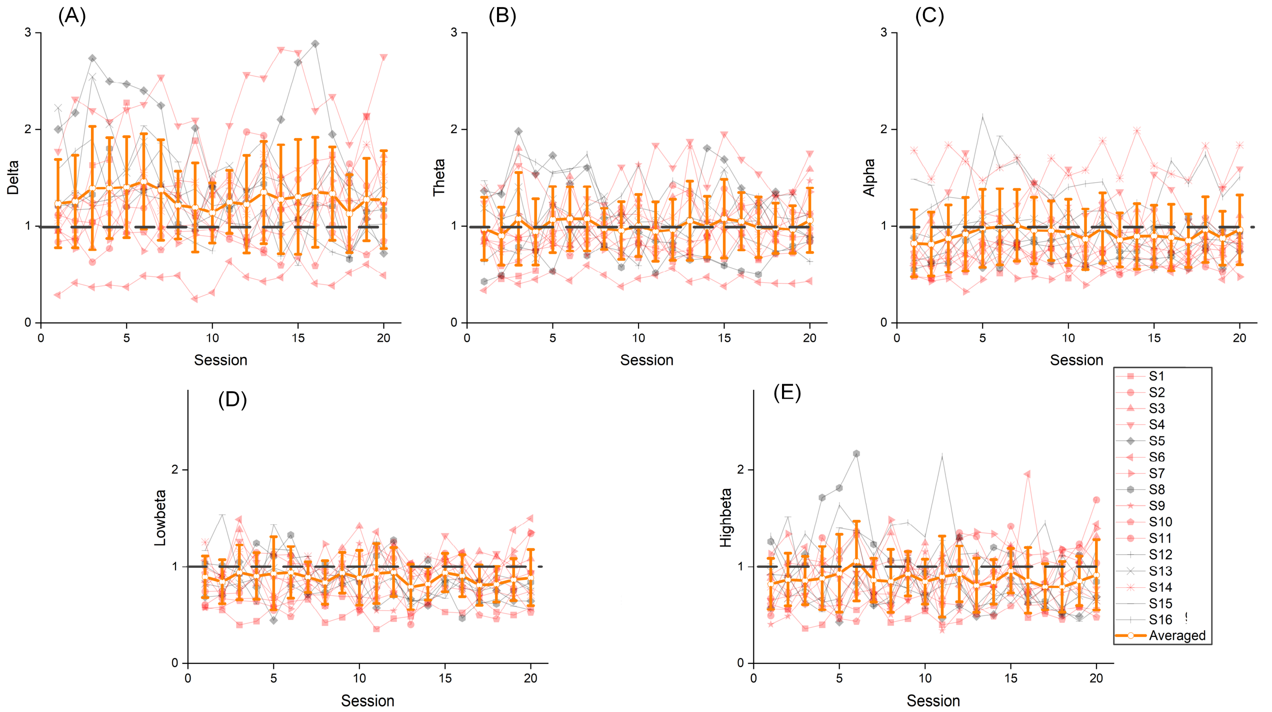

Fig. S1. Average ipsilesional task/rest EEG power variation during 20-sessions training. Lines and dots: data from each subject; Bold line and dots: averaged data of all subjects; Black and red: patients without or with effective recovery (reaching the MCID level).

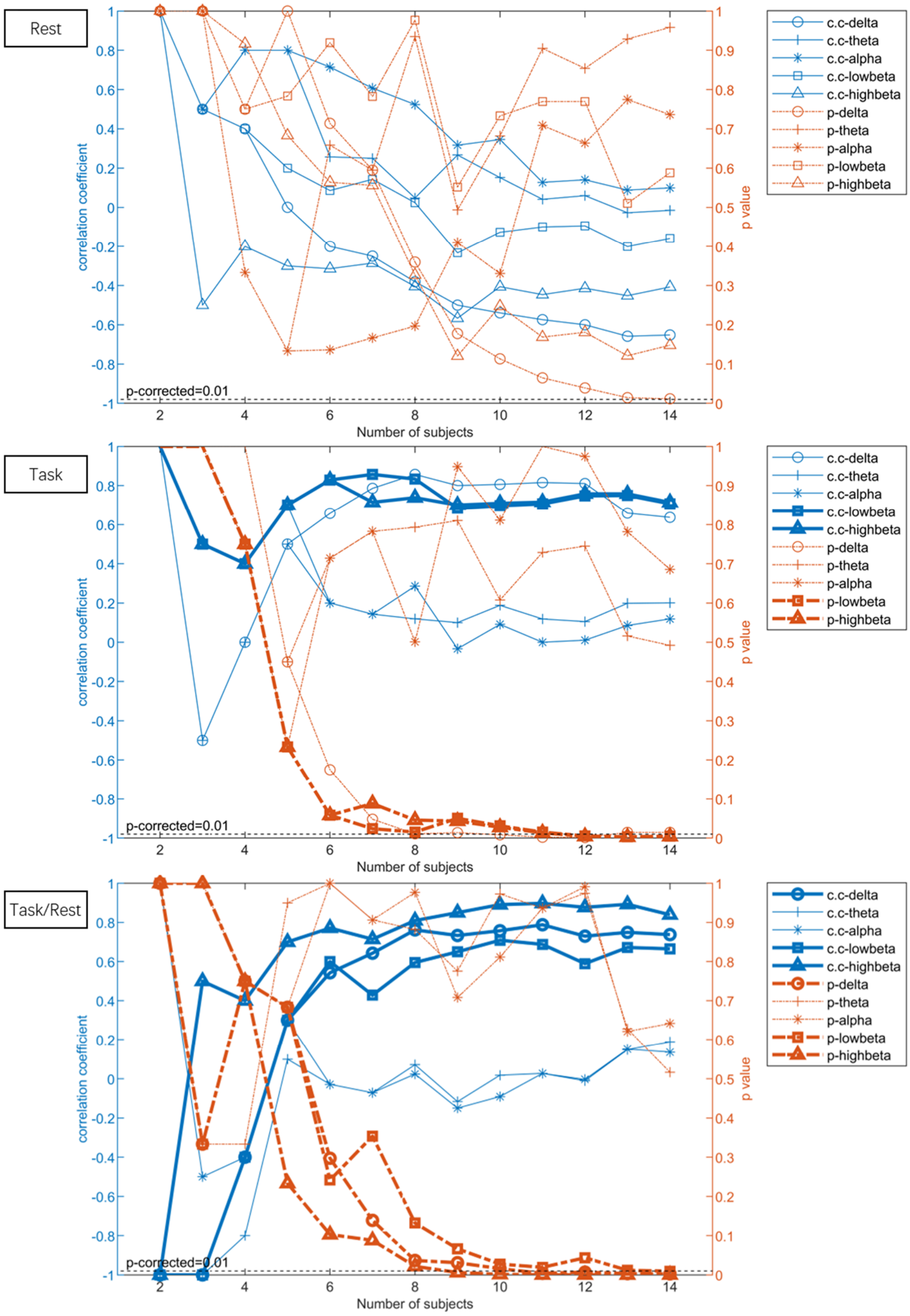

Fig. S2. The variation of correlation coefficient and p value (ΔEEG power vs ARAT improvements) as the function of number of subjects. Bold lines denote those values reaching significant level (p<0.01). The correlation coefficient and p value presented to be convergent with the increasing of subjects.

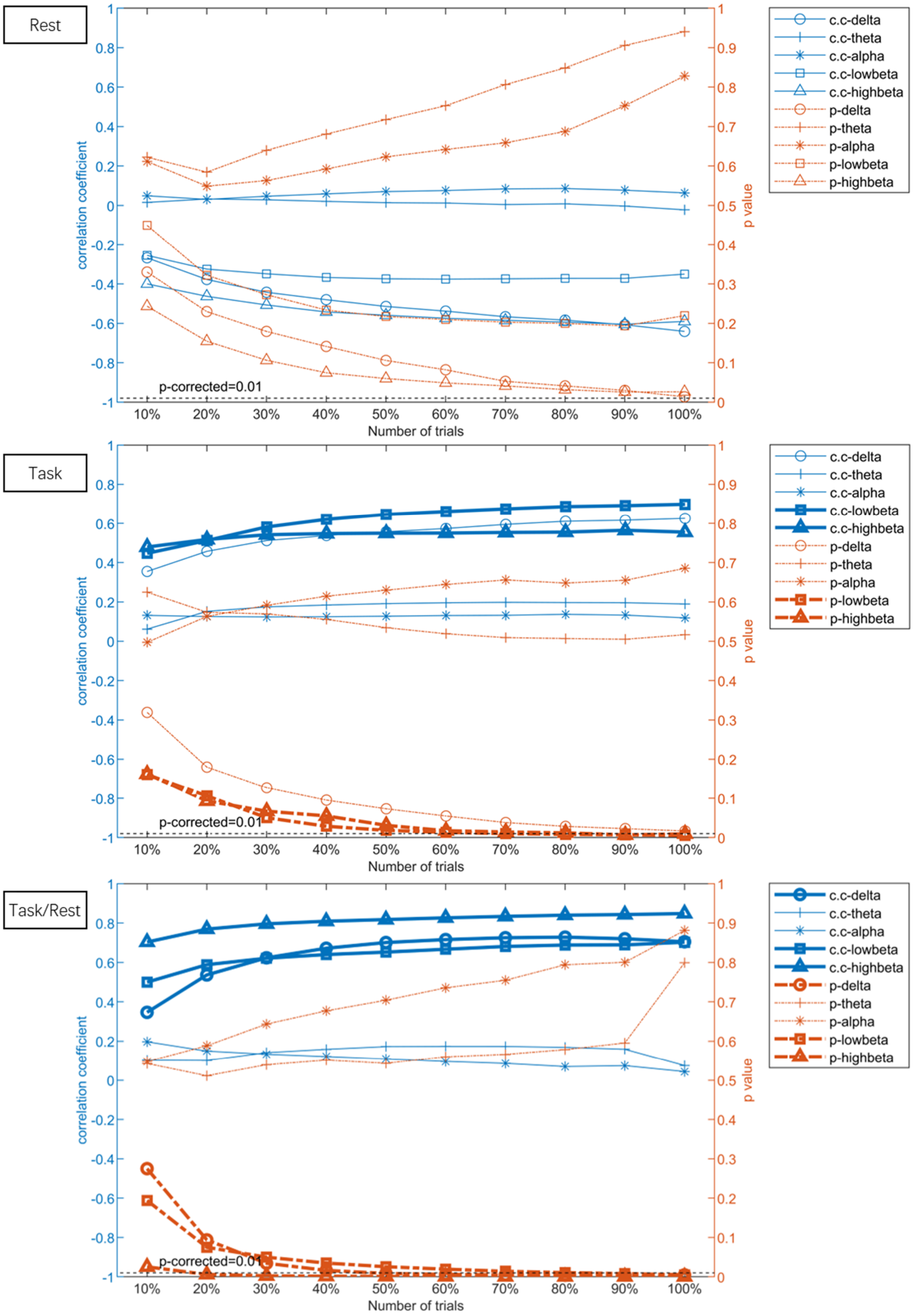

Fig. S3. The variation of correlation coefficient and p value (ΔEEG power vs ARAT improvements) as the function of number of trials proportions (from 10% to 100% each 10%). These data are averaged with the leave one subject out method (repeated 1000 times in random manner with the data of one subject out). Bold lines denote those values reaching significant level ($p<0.01$). The correlation coefficient and p value presented to be convergent with the increasing of trials.
